# The History of Minute Bloodvessels

**Published:** 1870-11

**Authors:** 


THE HISTORY OF MINUTE BLOODVESSELS.—Dr. J. J. Woodward, of the U. S. Army, has written a report to the Surgeon-General, on certain points in connection with this subject, which is published in the *Medical Record.* This paper is mainly a description of certain observations and experiments on the minute vessels, having a bearing on the theory of Prof. Cohnheim, and others, of the passage of white corpuscles through stomata in the walls of the vessels, in inflammation.

He describes eleven specimens, which he has prepared by staining with a dilute solution of nitrate of silver, all of which he illustrates by photo-micrographs, and in which he shows the form of cells, nuclei, and what seem to be the stomata.

If a solution of 1 part nitrate silver to 400 parts of water, be brushed over a clean epithelial surface, from a recently-killed animal, and the tissue then washed with distilled water, and exposed to the light, it will be found, on examination by the microscope, that a brownish-black pigment has been deposited on the boundaries of the epithelial cells, while the cells themselves are stained very little or not at all.

If the same solution is injected into a vessel, and it be afterward exposed to the light, the lining epithelium is plainly mapped out.

After the staining with the silver has been accomplished, a solution of carmine in borax will, by soaking the tissue in it, cause all the nuclei to become tinted red.

The cells lining the smallest vessels are shown by the specimens to be of an elongated outline, one four-hundreth to one five-hundreth in. long, and only about one-fifth as wide, with irregular zigzag borders. These borders interlock with one another, like the bones of the cranium forming the suturus, except where certain little openings are observed, which are presumed to be the stomata or openings through which white blood corpuscles may pass. These openings are to be observed most abundantly in veins of moderate size. They were found most numerous in veins one-fiftieth of an inch in diameter, or even larger, and they become smaller and rarer in smaller branches. They are comparatively infrequent in the capillaries, and still more so in the small arteries. In number and size, they vary in vessels of the same dimensions, in different parts of the body.

In figure they are rounded, oval, or oblong. "I have measured them as large as one-four thousandth of an inch in diameter, but smaller ones, one five-thousandth to one six-thousandth of an inch are more common, and the smallest and most frequent do not exceed one ten-thousandth of an inch." They are usually found in the marginal line, between adjacent epithelial cells. "From my study of these peculiar inter-cellular forms, I am inclined to regard with favor the opinion that they are actual openings in the epithelial layer."

It has been urged that, admitting that the stomata are veritable openings, the white corpuscles—one three-thousandth of an inch in diameter—cannot pass through them for want of room. But the extraordinary modification these little bodies undergo during their so-called "amœboid movements" would readily credit their capability of passing through such apertures. As the amœboid movement does not occur in the white corpuscles while rolling along in the torrent of the circulation, but only when the movement of the blood is arrested, more or less, completely, the fact that corpuscles do not habitually pass through the walls will not militate against the notion of palutous orifices. That corpuscles do pass out of the vessels is shown by a preparation from the stomach of a mare, dead of gastro-enteritis. In sections mounted with Canada balsam, many of the small veins were surrounded with white corpuscles, fixed in all steps of their amœboid movement. In a number of places, the white corpuscles can be observed in the interior of the vein, in the vascular wall, and in the adjacent connective tissue.

